# Development of a protocol testing the ability of *Stomoxys calcitrans* (Linnaeus, 1758) (Diptera: Muscidae) to transmit *Besnoitia besnoiti* (Henry, 1913) (Apicomplexa: Sarcocystidae)

**DOI:** 10.1007/s00436-012-3157-6

**Published:** 2012-10-12

**Authors:** E. Liénard, A. Salem, P. Jacquiet, C. Grisez, F. Prévot, B. Blanchard, E. Bouhsira, M. Franc

**Affiliations:** 1 Laboratoire de Parasitologie, Université de Toulouse, INP, ENVT, F-31076 Toulouse, France; 2 Adiagène, 38 rue de Paris, 22000 Saint-Brieuc, France

## Abstract

Cattle besnoitiosis due to the cyst-forming coccidian parasite *Besnoitia besnoiti* has recently been reported in expansion in Europe since the end of the twentieth century. The *B. besnoiti* life cycle and many epidemiological traits are still poorly known. Hematophagous flies, including the worldwide-distributed *Stomoxys calcitrans*, could be mechanical vectors in the contamination of mouthparts after the puncture of cutaneous cysts or ingestion of infected blood. In this study, a protocol is presented to assess more deeply the role of *S. calcitrans*, reared in laboratory conditions, in parasite transmission. A preliminary trial showed that stable flies could transmit tachyzoites from bovine artificially parasite-enriched blood to *B. besnoiti*-free blood using glass feeders. Evidence of transmission was provided by the detection of parasite DNA with Ct values ranging between 32 and 37 in the blood recipient. In a second time, a *B. besnoiti*-infected heifer harboring many cysts in its dermis was used as a donor of *B. besnoiti*. An interruption of the blood meal taken by 300 stable flies from this heifer was performed. Immediately after the blood meal was interrupted, they were transferred to a glass feeder containing *B. besnoiti*-free blood from a non-infected heifer. Quantitative PCR and modified direct fluorescence antibody test (dFAT) were used to detect *B. besnoiti* DNA and entire parasites, respectively, in the blood recipient, the mouthparts, and the gut contents of *S. calcitrans* at two time intervals: 1 and 24 h after the interrupted blood meal. Parasite DNA was detected at both time intervals (1 and 24 h) in all samples (blood recipient, mouthparts, and gut contents of stable flies) while entire parasites by dFAT were only found in the abdominal compartment 1 h after the interrupted blood meal. Then, *S. calcitrans* were able to carry *B. besnoiti* from chronically infected cattle to an artificial recipient in the conditions of the protocol.

## Introduction

*Besnoitia besnoiti* (Henry, 1913), a cyst-forming apicomplexan parasite, is the causative agent of cattle besnoitiosis. This disease, widely distributed in Africa, Asia, and in southwestern Europe, has spread in Portugal (Cortes et al. 2005), in Spain (Fernández-García et al. 2009), from southern France to central and western France since the end of the twentieth century (Alzieu et al. 2007) with recent and limited outbreaks in Germany (Mehlhorn et al. 2009) and Italy (Agosti et al. 1994) probably due to importation of infected cattle (Olias et al. 2011). Since 2010, cattle besnoitiosis is considered a reemerging disease according to the European Food Safety Authority (http://www.efsa.europa.eu/en/scdocs/doc/1499.pdf). Severe economic losses are observed in infected farms due to definitive or transient sterility in bulls, abortions, decline in milk production, mortality, and low body conditions, resulting in depreciating slaughter values and skin quality (Pols 1960; Cortes et al. 2005). The course of the disease is well described (Jacquiet et al. 2010). During the acute stage of the disease, tachyzoites multiply quickly within bovine macrophages and endothelial cells of blood vessels. The chronic stage is characterized by the formation of numerous cysts containing thousands of bradyzoites in various tissues including the skin. However, the life cycle of *B. besnoiti* and the routes of contamination are not yet clearly elucidated (Diesing et al. 1988; Kiehl et al. 2010; Basso et al. 2011; Olias et al. 2011). The cattle-to-cattle transcutaneous contamination is probably the most common way of infection via hematophagous insects. Experimentally, tachyzoites in blood or cutaneous bradyzoites have been successfully transmitted from an infected animal to a susceptible one by, respectively, the transfusion of large volumes of infected bovine blood or through the mechanical transmission by biting flies such as tabanids and *Stomoxys calcitrans* (Linnaeus, 1758) commonly named stable fly (Cuillé et al. 1936; Pols 1960; Bigalke 1968). Since the survey of Bigalke (1968) showing the mechanical vector role of those flies, these works, to our knowledge, have not been repeated to assess the epidemiological importance of *S. calcitrans*, one of the most serious pests of herds (Taylor et al. 2012), in the transmission of besnoitiosis. Mechanical transmission may occur when a stable fly is interrupted during blood feeding by host defensive behavior or other flies (Schofield and Torr 2002) and completes its blood meal on nearby animals (Doyle et al. 2011). A large proportion of animals exposed to and infected by *B. besnoiti* only become seropositive without developing clinical signs (Bigalke 1968; Jacquiet et al. 2010). Then, the use of an artificial host recipient, such as a blood glass feeder, to estimate the parasite carriage in *S. calcitrans* could eliminate the bias of cattle susceptibility (Doyle et al. 2011). The aim of this study was to establish a technical protocol to assess the role of *S. calcitrans*, fed with blood from a glass feeder, as a mechanical vector of *B. besnoiti* from infected cattle harboring cutaneous cysts.

## Material and methods

### Source and maintenance of *S. calcitrans*

A colony of stable flies is established at the ENVT (France) since May 2009. More details about rearing methods are provided by Salem et al. (2012). Adult stable flies required for the experimental purpose were 2 to 5 days old. They were fed only with water and honey ad libitum after emergence and before experiments. To study the ability of *B. besnoiti* to be transmitted, one or two sterilized glass feeders (according to the protocol used) were placed in contact with the upper side of the mesh cages (30 × 30 × 30 cm). A synthetic membrane (Parafilm 3M, Pechiney Plastic Packaging, Chicago, IL) sealed the inner chamber containing bovine blood of the glass feeder. Stable flies fed on blood by piercing the membrane through the mesh of the cage with their mouthparts.

### Source of *B. besnoiti*-free blood

The uninfected blood was provided by a 14-month-old heifer reared at the ENVT, which has not been treated with insecticides within 3 months prior to the study. The blood was collected in 4-ml tubes containing 60 USP U Lithium Heparin (Terumo Europe N.V., Leuven, Belgium) to prevent coagulation at the day of experiment.

### Source of *B. besnoiti-*infected blood and skin

The source of bradyzoites, used in transmission experiments 3 and 4 (see below), was a second 14-month-old heifer with chronic besnoitiosis. The infected heifer was reared at the ENVT under the same conditions as the uninfected cow. It came from an infected farm in Dordogne (France). Regarding both heifers, the presence or absence of *B. besnoiti* cutaneous cysts were checked clinically, and at least two biopsies (Biopsy Punch 8 mm, Kruuse, Langeskov, Denmark) were taken from the right forelimb and the neck. This was then followed by direct observation and quantitative PCR (see below). Their serological status against bovine besnoitiosis was also evaluated by Western blot (see below).

### Serological analysis of heifers

Purified *B. besnoiti* tachyzoites from a strain isolated in the French Pyrenees were used as antigen for the Western blot (WB) and to infect the blood in experiment 2 (see below). Vero cells and *B. besnoiti* tachyzoites are maintained at the ENVT since 2008 as described by Cortes et al. (2006a). WB procedures were adapted from Cortes et al. (2006a) and were performed as described by Liénard et al. (2011). A serum was considered positive if the three main antigenic domains (low, medium, and high molecular weights) were observed with a set of at least four bands within each of them (Cortes et al. 2006a).

### Experimental design

For all experiments, the sex ratio of *S. calcitrans* was close to 1:1. Experiments were achieved in November 2011 and in January 2012 when wild adult stable flies were not present in cattle facilities.

#### Experiment 1: control group

**Fig. 1:**
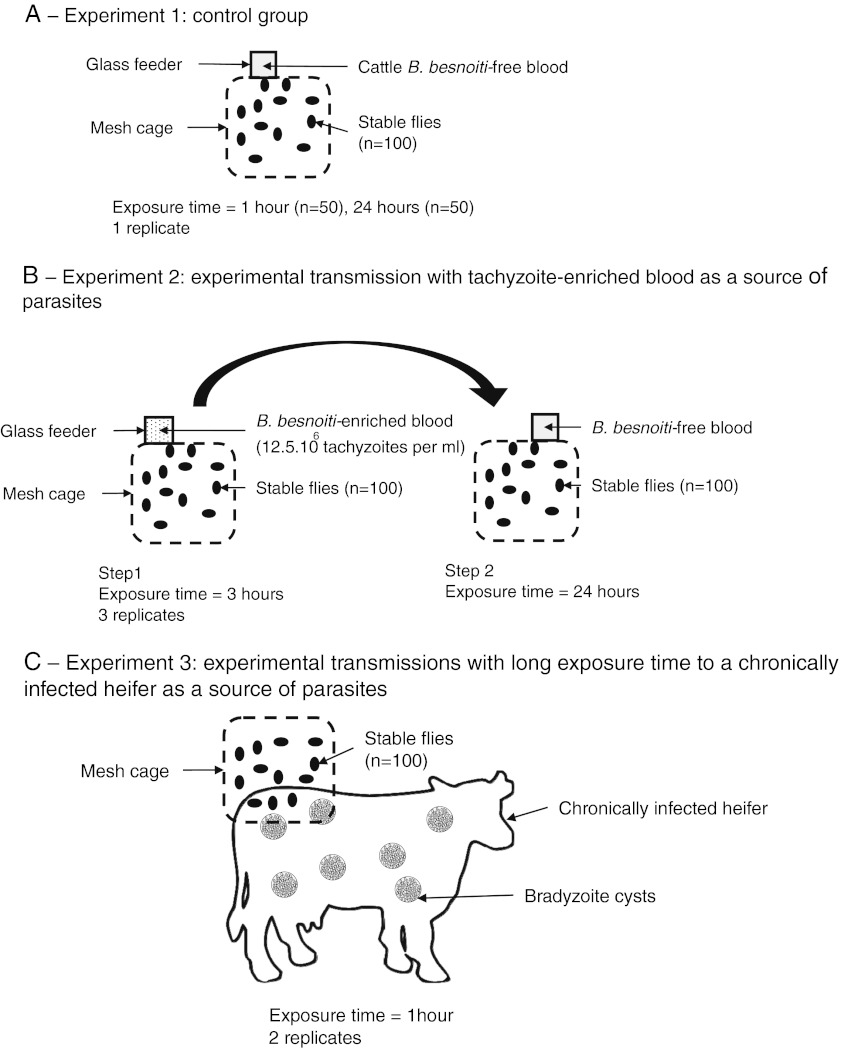

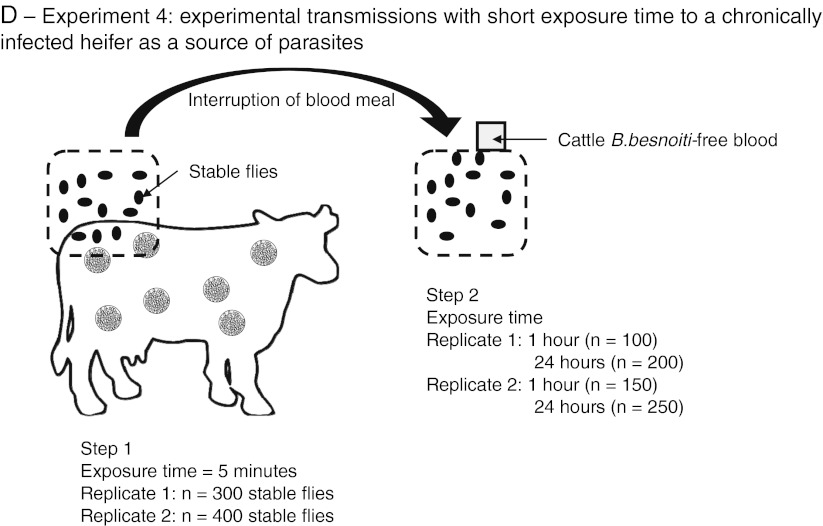
Diagrams of the experimental design. **a** Experiment 1: control group. **b** Experiment 2: experimental transmission with tachyzoite-enriched blood as a source of parasites. **c** Experiment 3: experimental transmissions with long exposure time to a chronically infected heifer as a source of parasites. **d** Experiment 4: experimental transmissions with short exposure time to a chronically infected heifer as a source of parasites

It was firstly necessary to assess whether some organisms present within *S. calcitrans* would interact with further analyses. Moreover, it was also necessary to confirm that laboratory-bred *S. calcitrans* were free from *B. besnoiti* contamination. Two control groups were then defined. A first group of 50 *S. calcitrans* were fed on 8 ml uninfected blood from the *B. besnoiti*-free heifer contained in a glass feeder during 1 h while a second group was exposed to the same blood for 24 h (Fig. 1a). At the end of the exposure, they were immediately knocked down at −20 °C for 1 h and dissected. For each group, mouthparts were manually removed under a magnifying glass with a needle and pooled into a grinding tube containing 1.4 ml of PBS (Bio-Rad, Marnes-la-Coquette, France). Abdomens of engorged flies were also opened with a needle, and abdominal contents were collected and pooled into the same sterile 4-ml tube containing 3 ml of PBS. Two tubes were eventually available for each control group. After mixing, both 3-ml tubes were equally divided to assess the presence of *B. besnoiti* by direct fluorescent antibody test (dFAT, entire parasites) and quantitative PCR (qPCR, parasite DNA). Blood samples of the *B. besnoiti*-free heifer before and after exposures (1 and 24 h) to *S. calcitrans* in glass feeders were also analyzed with both of these methods.

#### Experiment 2: experimental transmissions with tachyzoite-enriched blood as a source of parasites

This step was a preliminary trial of transmission before using a chronically infected heifer as a source of parasites. The aim was to assess the opportunity of *B. besnoiti* transmission from infected blood to uninfected blood by *S. calcitrans* using artificial tachyzoite-enriched blood as a source of parasites and recipient. A group of 100 stable flies was maintained in mesh cages (30 × 30 × 30 cm). A first double-chambered glass feeder was placed in contact with the upper side of the cage for 3 h. The inner chamber of this glass feeder contained 8 ml of blood from the uninfected heifer mixed with 10^8^ *B. besnoiti* culture tachyzoites. After 3 h, the first glass feeder was removed, and a second glass feeder with *B. besnoiti*-free blood from the same heifer was set up for 24 h (Fig. 1b). Quantitative PCR was used to detect parasite DNA in 2 ml of blood from both glass feeders (donor and recipient). This experiment was replicated three times.

#### Experiment 3: experimental transmissions with long exposure time to a chronically infected heifer as a source of parasites

The aim of this trial was to assess the ability of *S. calcitrans* to ingest *B. besnoiti* bradyzoites contained in cutaneous cysts via bites. A group of 100 stable flies were transferred into a 15 × 15 × 15-cm cage enclosed by thin wire mesh allowing them to bite after being placed on bovine skin. Sedative xylazine hydrochloride (0.1 mg/kg) (Rompun 2 %, Bayer Inc., Puteaux, France) was administered to the chronically infected heifer via intramuscular injection. A 20 × 20-cm^2^ area of skin on the right side of the rump was washed with water and shaved. An external antiseptic and antifungal solution of 10 % povidone iodine (Vétoquinol Vétédine Solution, Vétoquinol, Tarare, France) was applied and rinsed. The cage was manually maintained for 1 h (Fig. 1c). At the end, all stable flies were treated as previously described for the control groups (dFAT and qPCR). The test was replicated once more.

#### Experiment 4: experimental transmissions with short exposure time to a chronically infected heifer as a source of parasites

The aim of this trial was to assess the ability of *S. calcitrans* to transfer parasites from a chronically infected heifer to *B. besnoiti*-free bovine blood contained in an artificial glass feeder. A group of 300 stable flies was placed on the rump of the chronically infected heifer according to the same conditions as experiment 3. The duration of the heifer exposure was interrupted after 5 min (Dougherty et al. 1995). Immediately, all stable flies were allowed to achieve their blood meal on a glass feeder providing the *B. besnoiti*-free blood for 1 h (Fig. 1d). One hour later, 100 stable flies and 2 ml blood from the glass feeder recipient were removed to be treated as previously described for the control group. The remaining 200 stable flies were left to feed on the same bovine blood for 24 h before analyses already presented. Dead or non-blood-engorged flies were discarded. This trial was replicated once again with 400 *S. calcitrans* (150 flies after 1 h and 250 after 24 h).

### Detection of *B. besnoiti*

The dFAT was essentially achieved as described by Schares et al. (2010) and was adapted to detect entire parasites from the blood glass feeder, as well as the mouthparts and abdominal contents of stable flies. Briefly, before the suspension in a 4 % formaldehyde solution in PBS and the fixation on slides, red blood cells were hemolyzed by adding nine volumes of sterile water to one volume of blood during 5 min. Parasites were not crushed throughout this step. For each test, one immunofluorescence glass slide, to which were added eight drops, was prepared. Tachyzoites from culture were used as positive control slides. The parasite suspension was distributed in 15-μl drops on slides, dried at 37 °C, and fixed in ice-cold acetone (−20 °C) for 10 min. Moreover, a highly positive serum sample from a chronically infected cow (seven drops per slide) and a negative serum (one drop per slide) were used at 1:200 dilution. This dilution (1:200) was used as a positive cutoff value (Gentile et al. 2012). Rabbit anti-bovine IgG (whole molecule)–FITC (Sigma-Aldrich, Saint-Quentin Fallavier, France) was used as a conjugate at a 1:300 dilution according to the manufacturer's recommendations. The reading was performed under a fluorescence microscope at ×400 magnification (Axio Scope.A1, Carl Zeiss, Le Pecq, France). The presence of an entire parasite was confirmed when a complete and peripheral bright fluorescence of the parasite membrane was observed (Schares et al. 2010).

Quantitative PCR was used to detect *B. besnoiti* DNA from bovine skin and blood samples of the two heifers, from mouthparts and abdominal contents of *S. calcitrans* and from blood of glass feeders before and after transmission trials. Before DNA extraction with the QIAmp® DNA Mini Kit (Qiagen, Courtabœuf, France) commercial kit, mouthparts of stable flies were previously ground with the TeSeE™ Purification Kit (Bio-Rad, Marnes-la-Coquette, France) according to the manufacturer's recommendation. *B. besnoiti* ITS-1 amplification was performed with the commercial PCR kit AdiaVet™ Besnoitia (AES Chemunex, Bruz, France). The quantitative PCR was performed with the Stratagene MX3005P thermal cycler (Agilent Technologies, La Jolla, CA), and results were analyzed using the MxPro QPCR version 4.10 software (Agilent Technologies, La Jolla, CA). A threshold cycle (Ct) value of 40 corresponded to a negative result.

## Results

### Experiment 1.

The serum of the *B. besnoiti*-free heifer was negative for *B. besnoiti* infection as determined by WB. Its blood and skin biopsies were qPCR-negative. None of the *S. calcitrans* from both control groups that fed on the blood of this heifer were found positive by the *B. besnoiti*-specific qPCR (Table 1) or dFAT.

**Table 1:** Cycle threshold values (Ct) of *B. besnoiti* ITS-1 in *S. calcitrans* or in blood glass feeders according to experiments

Experiment	Replicate	Duration of exposure to		Number of stable flies used per replicate and per experiment	Ct of *B. besnoiti* ITS-1		
		Skin of *B. besnoiti-*infected heifer	Blood of *B. besnoiti*-free heifer		In *S. calcitrans*		In recipient blood from glass feeder
					Mouthparts	Abdominal contents	
Experiment 1	1	None	1 h	50	No Ct	No Ct	No Ct
			24 h	50	No Ct	No Ct	No Ct
Experiment 3	1	1 h	None	100	39	34	N.D.
	2			100	36	24	N.D.
Experiment 4	1	5 min	1 h	100	35	29	34
			24 h	200	39	34	32
	2		1 h	150	39	27	No Ct
			24 h	250	No Ct	33	39

Results of experiment 2 were reported directly in the text

*No Ct* negative Ct values are ≥40; *N.D.* not determined

### Experiment 2.

This trial showed that *S. calcitrans* transmitted *B. besnoiti* DNA from highly infected blood to uninfected blood. Indeed, Ct values for the glass feeder containing tachyzoite-enriched blood were very low in all three replicates (16 and twice 17), which was to be expected. Parasite DNA was detected in the glass feeder recipient with Ct values of 32 and twice 37 after a 24-h exposure. This checking step allowed the following experiments involving a living heifer acting as a source donor.

### Experiment 3.

The serum of the chronically infected heifer was clearly positive by WB. Parasite DNA was detected in blood (Ct value of 33) and in two skin biopsy punches from the neck and the forelimb with Ct values of 16 and 18, respectively. Experiment 3 showed that stable flies could ingest parasites from cutaneous cysts after 1 h of exposure. qPCR tests (Table 1) were positive in both replicates with a higher Ct value for the mouthparts than that of the abdominal contents. The dFAT was only positive for the gut contents of stable flies.

### Experiment 4.

**Fig. 2:**
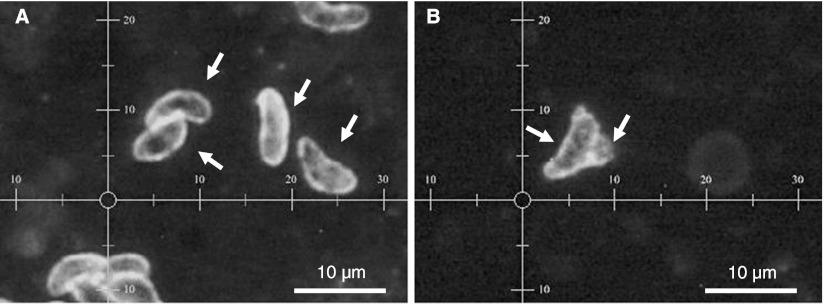
Positive dFAT for tachyzoite culture (**a**) and for abdominal contents of *S. calcitrans* (**b**) after 1-h exposure on blood glass feeder preceded by 5 min of exposure to the heifer's skin (experiment 4). White rows indicate *B*. *besnoiti* parasites

DNA was detected in the intestinal contents and mouthparts of *S. calcitrans* 1 h after the blood meal was interrupted. The Ct values of abdominal contents were lower than Ct values of mouthparts (Table 1). Twenty-four hours after feeding on the infected heifer, the Ct values of the abdominal contents of stable flies were higher (34 and 33 according to the replicates) than those obtained after 1-h feeding (29 and 27, respectively, Table 1). No Ct and a very high value (39) were recorded from mouthparts 24 h after feeding according to replicates 1 and 2. The blood in the glass feeder was positive in the first replicate only, at 1 h (Ct = 34) and 24 h (Ct = 32) of exposure. Results of dFAT revealed some discrepancy with qPCR. *B. besnoiti* tachyzoites isolated from Vero cell culture were clearly observed on the control slides (Fig. 2a). Entire parasites were clearly identified in the abdominal contents of the stable flies (Fig. 2b). No entire *B. besnoiti* could be observed by dFAT on the slides in the mouthparts nor in the blood from the glass feeder after any of the exposure times (experiment 3 and 4).

## Discussion

Cattle besnoitiosis has been neglected until its recent geographic expansion in Europe (Olias et al. 2011). Some strains have been isolated in various European countries: Portugal (Cortes et al. 2006b), Germany (Schares et al. 2009), Spain (Fernández-García et al. 2009), Italy (Gentile et al. 2012), and for the first time in France.

The contamination of mouthparts and abdominal contents of stable flies was achieved with our protocol using a living and chronically infected heifer as a source of *B. besnoiti*. The use of laboratory colonies of stable flies has the advantage of preventing previous *B. besnoiti* infections, as also demonstrated by Bigalke (1968). It was about the 15th generation of stable flies that was used in November 2011 (Salem et al. 2012), and no evidence has been collected showing that *B. besnoiti* could pass through the eggs of stable flies (Bigalke 1968).

The use of glass feeders with a thin membrane was also efficient for studying the transmission ability of *S. calcitrans* by using an artificial and highly tachyzoite-enriched blood or chronically infected cattle as a source of parasites. In experiment 2, the parasite burden in the blood was significantly high, which increases the probability of ingestion by *S. calcitrans* since no data were previously available. Moreover, this level of infection most likely did not occur naturally. As shown with experiments 3 and 4, stable flies were able to ingest parasites while blood feeding on chronically infected cattle, which has never been demonstrated since the survey of Bigalke (1968). Using qPCR for experiment 4, we did not observe a decrease in Ct values between 1 and 24 h after exposure in the abdominal contents of stable flies, suggesting that no effective parasite multiplication had occurred within the gut of stable flies. Conversely, the increase of Ct values could imply the death of parasites and destruction of parasite DNA. One possible explanation could be the presence of stomoxyn, a defense helical peptide of 42 amino acids secreted by the anterior part of the gut after the blood meal, which demonstrated a trypanolytic activity (Boulanger et al. 2002). Stomoxyn probably had a wide range of antimicrobial activities (Boulanger et al. 2002) that could also include *B. besnoiti*, given that no entire parasite was observed by dFAT after 24 h in the abdominal contents of stable flies.

The parasite burden in the mouthparts was probably very low. The volume of blood retained in the mouthparts of *S. calcitrans* was close to 0.4 nl (Scoles et al. 2005), which is smaller than the volume of blood contained in the tabanids' mouthparts evaluated at 12.5 nl (Scoles et al. 2008). Bigalke (1968) estimated that 52,000 to 292,500 stable fly bites were required to cause infection in cattle whereas only three horse fly bites were sufficient. Ct values of mouthparts for both experiments 3 and 4 were very high, which highlighted the presence of a low burden of parasite DNA. Indeed, dFAT analysis on this stable fly compartment remained negative in both experiments. The survival time of a parasite in the mouthparts of *S. calcitrans* is probably very short, as suggested by Bigalke (1968), who did not obtain successful contaminations with stable flies 3 h after their interrupted and infected blood meal. Firstly, some *B. besnoiti* parasites could have been destroyed by some components of *S. calcitrans* saliva (Scoles et al. 2005). Secondly, the adapted dFAT probably suffered from low sensitivity. Those facts could also explain why no entire parasite was detected by dFAT in the blood of the glass feeder in experiment 4. However, parasite DNA was detected by qPCR and turned out to be positive in recipient blood, suggesting that it was possible to transfer a parasite from a living donor to another host, either by proboscis flushing or by regurgitation (Doyle et al. 2011).

Non-hematophagous arthropods, e.g., *Musca domestica* and *Musca autumnalis*, which feed on lachrymal secretions, on blood from skin injuries or on other exudative liquids, have been suspected to transmit *B. besnoiti* mechanically. This has never been confirmed (Bigalke 1968). In this case, potential routes of mechanical transmission may include parasite transfer from wounds of infected animals to wounds of susceptible ones, for example by direct contact of legs, exoskeleton, and licking mouthparts of insects, but this could be probably extremely rare in field conditions.

To conclude, since the unique survey of Bigalke (1968), we have collected some evidence with help of modern tools of parasite detection and proved that *S. calcitrans* could ingest *B. besnoiti* parasites from a chronically infected heifer harboring cutaneous cysts and could mechanically transmit these parasites to a uniform recipient substrate. Several improvements can be done on our protocol. For example, the increase of the number of stable flies implied in these transmission experiments to 500 or 1,000 could allow the detection of viable parasites by dFAT and decrease Ct values. Moreover, in epidemiological surveys, this protocol could also be applied to discriminate cattle that can be “suitable donors” of *B. besnoiti* (Bigalke 1968) among a population of seropositive but asymptomatic animals. In other words, it would be interesting to estimate the correlation between the Ct values obtained from the skin biopsy and the presence or absence of parasite and/or parasite DNA in the artificial recipient after the insects' interrupted blood meals.

## References

[CR1] Agosti M, Belloli A, Morini M, Vacirca G (1994). Segnalazione di un focolaio di Besnoitiosi in bovini da carne importati. Praxis Vet.

[CR2] Alzieu JP, Cortes HC, Gottstein B, Jacquiet P, Dorchies P, Schelcher F, L’Hostis M (2007) La besnoitiose bovine: actualités épidémiologiques et diagnostiques. Bull GTV Hors-série parasitisme des bovins : 41-–49

[CR3] Basso W, Schares G, Gollnick NS, Rütten M, Deplazes P (2011). Exploring the life cycle of *Besnoitia besnoiti*—experimental infection of putative definitive and intermediate host species. Vet Parasitol.

[CR4] Bigalke RD (1968). New concepts on the epidemiological features of bovine besnoitiosis as determined by laboratory and field investigations. Onderstepoort J Vet Res.

[CR5] Boulanger N, Munks RJL, Hamilton JV, Vovelle F, Brun R, Lehane MJ, Bulet P (2002). Epithelial innate immunity. A novel antimicrobial peptide with antiparasitic activity in the blood-sucking insect *Stomoxys calcitrans*. J Bio Chem.

[CR6] Cortes HCE, Leitao A, Vidal R, Vila-Vicosa MJ, Ferreira ML, Caeiro V, Hjerpe CA (2005). Besnoitiosis in bulls in Portugal. Vet Record.

[CR7] Cortes HCE, Nunes S, Reis Y, Staubli D, Vidal R, Sager H, Leitao A, Gottstein B (2006). Immunodiagnosis of *Besnoitia besnoiti* infection by ELISA and Western blot. Vet Parasitol.

[CR8] Cortes HCE, Reis Y, Waap H, Vidal R, Soares H, Marques I, da Fonseca IP, Fazendeiro I, Ferreira ML, Caeiro V, Shkap V, Hemphill A, Leitao A (2006). Isolation of *Besnoitia besnoiti* from infected cattle in Portugal. Vet Parasitol.

[CR9] Cuillé J, Chelle PL, Berlureau F (1936). Transmission expérimentale de la maladie dénommée “Sarcoporidiose cutanée” du boeuf (Besnoit et Robin) et déterminée par “*Globidium besnoiti*”. Bull Acad Med.

[CR10] Diesing L, Heydorn AO, Matuschka FR, Bauer C, Pipano E, Dewaal DT, Potgieter FT (1988). *Besnoitia besnoiti*: studies on the definitive host and experimental infections in cattle. Parasitol Res.

[CR11] Dougherty CT, Knapp FW, Burrus PB, Willis DC, Cornelius PL (1995). Behaviour of grazing cattle exposed to small population of stable flies (*Stomoxys calcitrans* L.). App Anim Behav Sci.

[CR12] Doyle MS, Swope BN, Hogsette JA, Burkhalter KL, Savage HM, Nasci RS (2011). Vector competence of the stable fly (Diptera: Muscidae) for West Nile Virus. J Med Entomol.

[CR13] Fernández-García A, Risco-Castillo V, Pedraza-Díaz S, Aguado-Martínez A, Álvarez-García G, Gómez-Bautista M, Collantes-Fernández E, Ortega-Mora LM (2009). First Isolation of *Besnoitia besnoiti* from a chronically infected cow in Spain. J Parasitol.

[CR14] Gentile A, Militerno G, Schares G, Nanni A, Testoni S, Bassi P, Gollnick NS (2012). Evidence for bovine besnoitiosis being endemic in Italy—first in vitro isolation of *Besnoitia besnoiti* from cattle born in Italy. Vet Parasitol.

[CR15] Jacquiet P, Liénard E, Franc M (2010). Bovine besnoitiosis: epidemiological and clinical aspects. Vet Parasitol.

[CR16] Kiehl E, Heydorn AO, Schein E, Al-Rasheid KAS, Selmair J, Abdel-Ghaffar F, Mehlhorn H (2010). Molecular biological comparison of different *Besnoitia* species and stages from different countries. Parasitol Res.

[CR17] Liénard E, Salem A, Grisez C, Prévot F, Bergeaud JP, Franc M, Gottstein B, Alzieu JP, Lagalisse Y, Jacquiet P (2011). A longitudinal study of *Besnoitia besnoiti* infections and seasonal abundance of *Stomoxys calcitrans* in a dairy cattle farm of southwest France. Vet Parasitol.

[CR18] Mehlhorn H, Klimpel S, Schein E, Heydorn AO, Al-Quraishy S, Selmair J (2009). Another African disease in Central Europa: besnoitiosis of cattle. I. Light and electron microscopical study. Parasitol Res.

[CR19] Olias P, Schade B, Mehlhorn H (2011). Molecular pathology, taxonomy and epidemiology of *Besnoitia* species (Protozoa: Sarcocystidae). Infect Genet Evol.

[CR20] Pols JW (1960). Studies on bovine besnoitiosis with special reference to the aetiology. Onderstepoort J Vet Res.

[CR21] Salem A, Franc M, Jacquiet P, Bouhsira E, Liénard E (2012) Feeding and breeding aspects of *Stomoxys calcitrans* (Diptera: Muscidae) under laboratory conditions. Parasite (in press) 10.1051/parasite/2012194309 PMC3671465 23193515

[CR22] Schares G, Basso W, Majzoub M, Cortes HCE, Rostaher A, Selmair J, Hermanns W, Conraths FJ, Gollnick NS (2009). First in vitro isolation of *Besnoitia besnoiti* from chronically infected cattle in Germany. Vet Parasitol.

[CR23] Schares G, Basso W, Majzoub M, Rostaher A, Scharr JC, Langenmayer MC, Selmair J, Dubey JP, Cortes HC, Conraths FJ, Gollnick NS (2010). Comparative evaluation of immunofluorescent antibody and new immunoblot tests for the specific detection of antibodies against *Besnoitia besnoiti* tachyzoites and bradyzoites in bovine sera. Vet Parasitol.

[CR24] Schofield S, Torr SJ (2002). A comparison of the feeding behaviour of tsetse and stable flies. Med Vet Entomol.

[CR25] Scoles GA, Lysyk TJ, Broce AB, Palmer GH (2005). Relative efficiency of biological transmission of *Anaplasma marginale* (Rickettsiales: Anaplasmataceae) by *Dermacentor andersoni* Stiles (Acari: Ixodidae) compared to mechanical transmission by *Stomoxys calcitrans* (L.) (Diptera: Muscidae). J Med Entomol.

[CR26] Scoles GA, Miller JA, Foil LD (2008). Comparison of the efficiency of biological transmission of *Anaplasma marginale* (Rickettsiales: Anaplasmataceae) by *Dermacentor andersoni* Stiles (Acari: Ixodidae) with mechanical transmission by the horse fly, *Tabanus fuscicostatus* Hine (Diptera: Muscidae). J Med Entomol.

[CR27] Taylor DB, Moon RG, Mark DR (2012). Economic impact of stable flies (Diptera: Muscidae) on dairy and beef cattle production. J Med Entomol.

